# Bioengineering of non-pathogenic *Escherichia coli* to enrich for accumulation of environmental copper

**DOI:** 10.1038/s41598-020-76178-z

**Published:** 2020-11-23

**Authors:** Dharmender K. Gahlot, Nayyer Taheri, Dhani Ram Mahato, Matthew S. Francis

**Affiliations:** 1 Department of Biology, University of York, Wentworth Way, York, YO10 5DD UK; 2 Department of Molecular Biology, Umeå University, 90187 Umeå, Sweden; 3 Department of Chemistry, Umeå University, 90187 Umeå, Sweden

**Keywords:** Applied microbiology, Environmental microbiology, Environmental biotechnology, Microbiology, Environmental sciences

## Abstract

Heavy metal sequestration from industrial wastes and agricultural soils is a long-standing challenge. This is more critical for copper since copper pollution is hazardous both for the environment and for human health. In this study, we applied an integrated approach of Darwin’s theory of natural selection with bacterial genetic engineering to generate a biological system with an application for the accumulation of Cu^2+^ ions. A library of recombinant non-pathogenic *Escherichia coli* strains was engineered to express seven potential Cu^2+^ binding peptides encoded by a ‘synthetic degenerate’ DNA motif and fused to Maltose Binding Protein (MBP). Most of these peptide-MBP chimeras conferred tolerance to high concentrations of copper sulphate, and in certain cases in the order of 160-fold higher than the recognised EC_50_ toxic levels of copper in soils. UV–Vis spectroscopic analysis indicated a molar ratio of peptide-copper complexes, while a combination of bioinformatics-based structure modelling, Cu^2+^ ion docking, and MD simulations of peptide-MBP chimeras corroborated the extent of Cu^2+^ binding among the peptides. Further, in silico analysis predicted the peptides possessed binding affinity toward a broad range of divalent metal ions. Thus, we report on an efficient, cost-effective, and environment-friendly prototype biological system that is potentially capable of copper bioaccumulation, and which could easily be adapted for the removal of other hazardous heavy metals or the bio-mining of rare metals.

## Introduction

Copper is an essential trace element that is important in cellular physiology in very low amount (up to 0.3 µM)^1,2^. It positively effects numerous cellular enzymes involved in energy metabolism. For examples, oxidoreductase and transferase enzymes utilise copper (in nM quantity) as a cofactor to facilitate electron transfer in redox reactions involved in metabolism and bioenergetics^3^. Therefore, organisms have evolved sophisticated resistance mechanisms to maintain copper homeostasis and counteract exposure to higher copper concentrations^4,5^.

Anthropogenic activities have elevated global copper concentration to over 3 μM, way beyond the current environmental quality standard (i.e.: currently set at 0.07 μM for open waters). This has negative repercussions for both marine and terrestrial life^6–9^. It is made even more alarming given that copper pollution in the environment has become commonplace^6–13^.

There is now an urgent need to develop a system for the efficient, cost effective and environment friendly remediation of copper. Detoxification of heavy metal pollution can occur with physio-chemical and biological methods^14–17^. Unlike physio-chemical methods that are uneconomic and generate large amounts of chemical waste, biological methods are eco-friendly and offer high specificity in the elimination and counteraction of desired heavy metals^17–20^. Moreover, counteraction of heavy metals using microorganisms, known as bioremediation, is cost-effective and can even provide a more permanent solution compared to other known methods^17–19^.

Bacteria expressing metal binding proteins have a range of applications that include bioremediation of toxic heavy metals, the bio-adsorption and recovery of rare metals, and in energy generation^21^. Recombinant DNA technology offers methods for anchoring metal binding peptide(s) to fusion partners such as bacterial outer membrane proteins^22^, lipopolysaccharide^23^, and the Maltose Binding Protein (MBP)^24^. For such fusions, size and amino acid composition of the cargo (poly)peptide can significantly affect the final topology of the generated chimera. For this reason, use of MBP as a fusion partner is attractive because it has solubilising properties that can promote proper folding of the fused (poly)peptide into its biologically active form^25^.

In this study, we designed a ‘synthetic degenerate’ DNA fragment of 30 base pairs, and which was expressed as a fusion at the C-terminal of MBP by harmless laboratory *E. coli* to generate a bank of semi-random peptides all of 9 amino acids length. Those predicted to contain a potential U-shaped Cu^2+^ ion coordination pocket conferred to bacteria resistance to copper that was about 160-fold higher than the recognised EC_50_ toxic levels of copper in soils^11^. Hence, this study demonstrates a simple, robust and innovative biological system for the efficient and effective binding of Cu^2+^ ions. We also suggest that this is a generally applicable approach for the bioremediation of various toxic heavy metals and for the bio-mining of rare metals.

## Results

### Crafting a small bank of MBP-fused semi-random peptides

**Figure 1:**
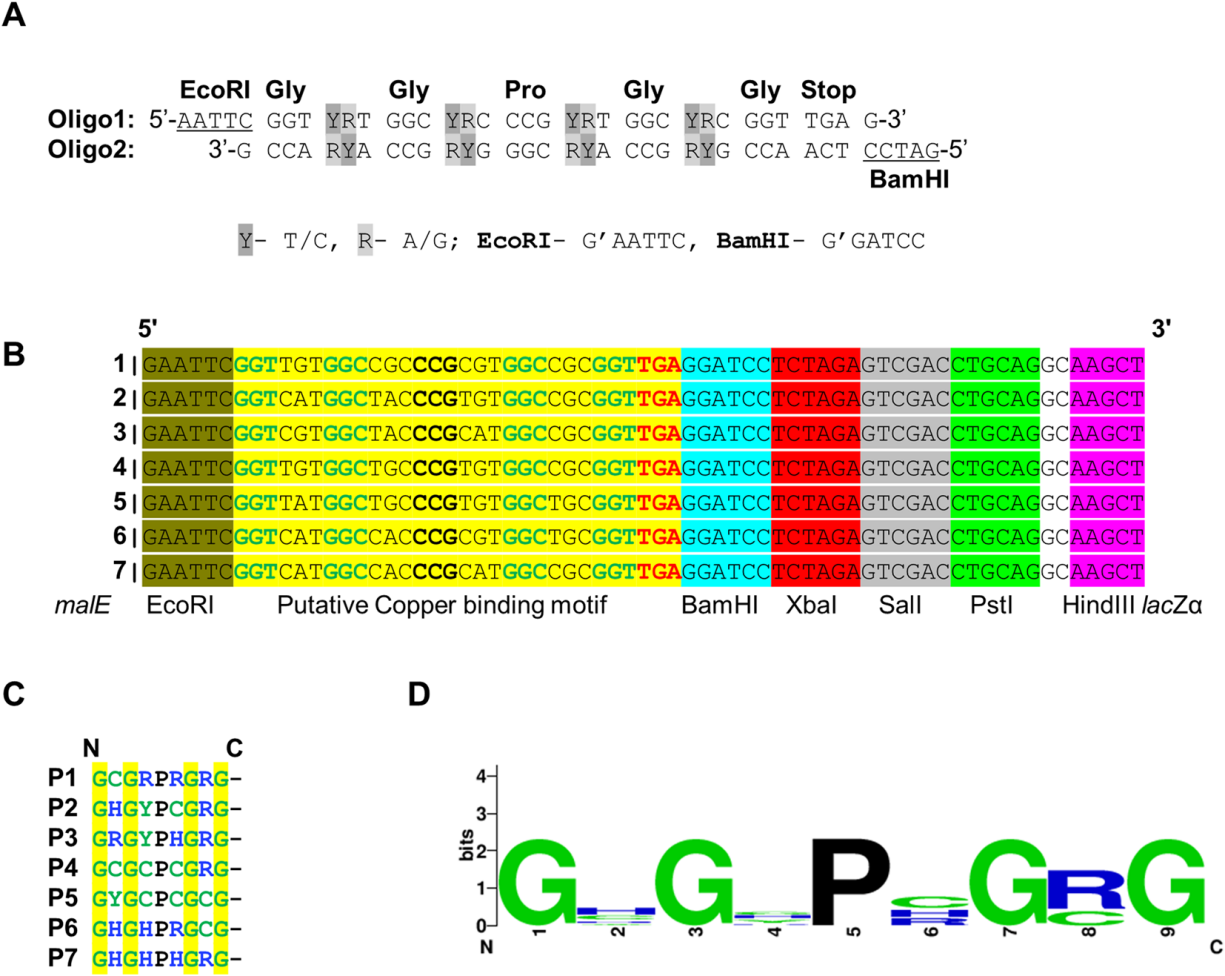
Crafting a small bank of MBP-fused semi-random peptides. (**A**) Two DNA oligos (Oligo1 and 2) were designed with nucleotides degeneracy (highlighted in grey) in order to code for potential metal binding residues, Cys, His, Arg and Tyr, distributed randomally, at the site of degeneracy, with an invariant Gly spacer and Pro at the centre. Restriction enzyme sites, EcoRI and BamHI (underlined) were incorporated at respective termini to facilitate the cloning of the anneled dsDNA motif into the pMAL-p2x plasmid to express *malE*-metal binding peptide fusions (chimeric MBP, cMBP). (**B.** Sequenced plasmids from 7 random recombinant *E. coli* strains encoded a cMBP fusion (cMBP1-7), containing putative Cu^2+^ binding motifs (yellow) flanked by BamHI and EcoRI restriction sites. (**C**) Translated putative Cu^2+^ binding motifs (yellow), encoding peptides 1–7 (P1-P7). As expected all encoded peptides contain a central Pro amino acid (P), Gly (G) spacer at alternative positions of the intended potential metal binding residues, Cys (C), His (H), Arg (R) and Tyr (Y) amino acids, and all are terminated by stop codon (−).(**D)** The consensus signature, Gly-X-Gly-X-Pro-X-Gly-Arg/Cys-Gly for P1-P7 peptides.

The aim of this study was to engineer harmless non-pathogenic bacteria with potential to accumulate copper. An earlier study with peptides rich in either His or Cys residues and fused to LamB showed variable and modest bioaccumulation capacity for Cu^2+^, and with little specificity^26^. We capitalised on this experience by creating a small bank of semi-random peptides in which every peptide encompassed a potential Cu^2+^ binding pocket formed by a random distribution of Cys, His, Arg and Tyr residues, and with an invariant Pro hinge and intermittent Gly spacer to facilitate Cu^2+^ co-ordination. To accommodate these new design features aimed at enhancing Cu^2+^ binding affinity, a minimum peptide length of 9 amino acids was required. To achieve this, nucleotide degeneracy was inbuilt into two complementary DNA oligonucleotides of 30 bases (Fig. 1A). With a unique restriction enzyme recognition site at their termini, the annealed duplex DNA motif was cloned in-frame into EcoRI and BamHI sites at the 3′ end of *malE* (encoding MBP) within the pMAL-p2x plasmid*.* The recombinant expression vector carried the chimeric *malE-*synthetic degenerate*-*dsDNA motif fusion under the control of the strong IPTG-inducible P_tac_ promoter. We used fusion to MBP to anchor the metal binding peptides, promote their extracytoplasmic targeting, and to facilitate their purification from the bacterial periplasm for eventual biochemical studies. Critically, MBP is a fusion of choice for this role because it rarely interferes with the proper folding of the cargo (poly)peptide^25,27^.

To demonstrate proof-of-concept, we sequenced seven randomly selected plasmids purified from well-isolated colonies of newly transformed *E. coli*. In all cases the sequence confirmed *malE*-fused to degenerate DNA motifs (Fig. 1B), with the latter having a small open reading frame encoding a putative metal binding peptide of 9-amino acids in length (Fig. 1C). As expected, predicted peptide sequence positioned a Pro amino acid in the centre, with randomly distributed Cys, His, Arg and Tyr amino acids throughout that were deliberately separated by a Gly spacer placed at every alternative position (Fig. 1C). From this analysis we inferred a consensus Gly-X-Gly-X-Pro-X-Gly-Arg/Cys-Gly signature for the identified peptides (Fig. 1D).

### Genetically engineered *E. coli* acquired resistance to copper

**Figure 2:**
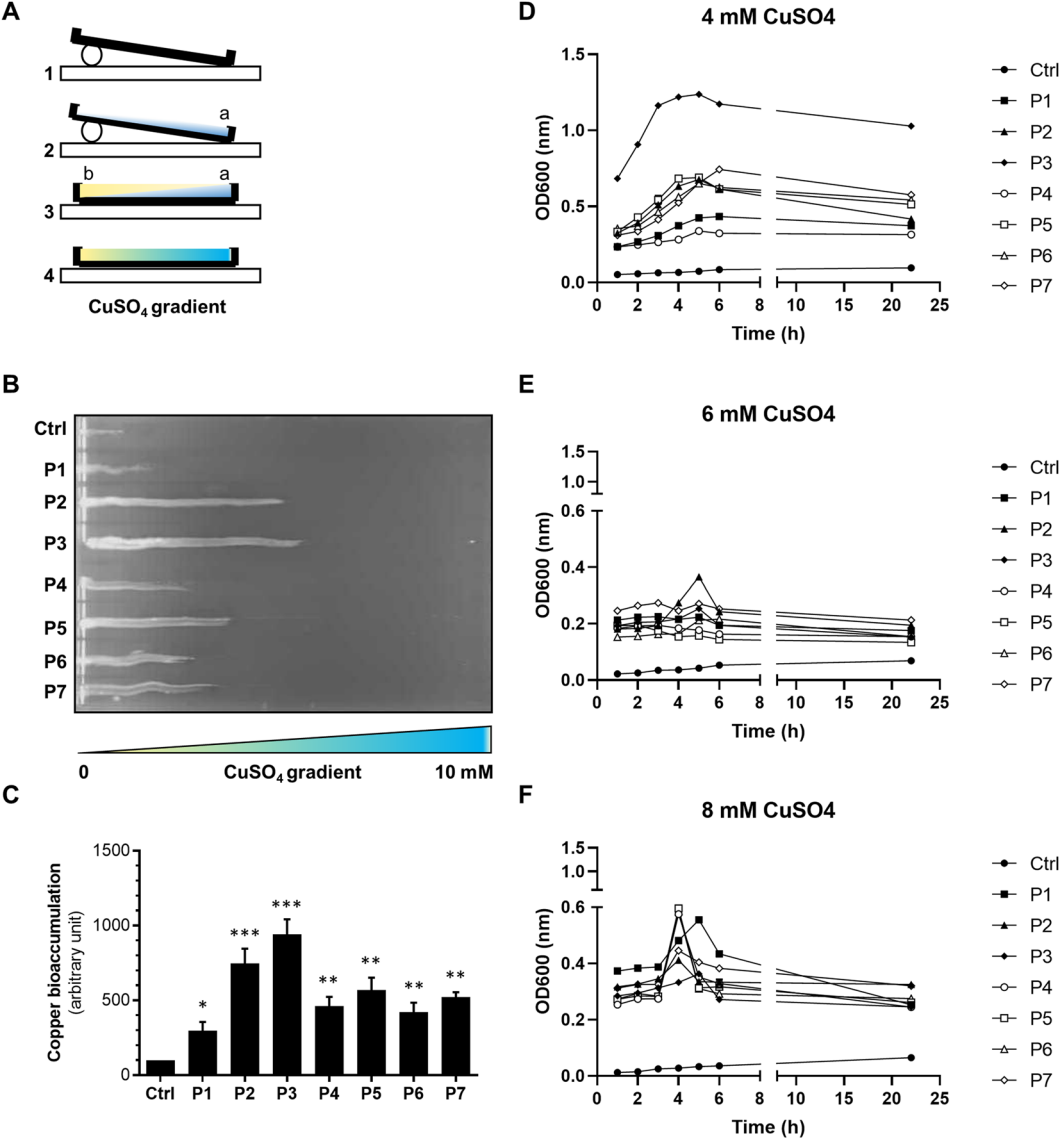
Resistance to Cu^2+^ by genetically engineered non-pathogenic *E. coli*. (**A**) Scheme of copper sulphate linear gradient in a sterile squared petri plate used to test copper resistance by the recombinant *E. coli* cells expressing MBP alone or cMBP fusions. In laminar airflow, sterile square petri plates were elevated from one side as depicted (step 1). Plates laying on the diagonal were first filled with molten LB agar, supplemented with ampicillin, IPTG and copper sulphate (step 2, a). After being allowed to cool, plates were laid flat and an equal volume of molten LB supplemented only with ampicillin and IPTG was overlaid (step 3, b). The plates formed a copper sulphate linear gradient (step 4). (**B**) Capacity of recombinant *E. coli* cells expressing MBP alone (Ctrl) or an individual MBP-peptide fusion (peptides P1 to P7) to resist copper as measured by growth on copper sulphate gradient agar. Standardised bacterial inoculums were streaked on gradient plates in the direct of low to high copper sulphate gradient and incubated at 37 °C overnight. A representative plate is shown. The original plate image is shown in the Supplementary data Fig. S1. (**C**) ImageJ-quantification of copper resistance capacity of recombinant *E. coli* strains. Data presented as the mean ± SEM for three independent biological replicates, with all three performed in triplicate. Statistical difference was calculated with one-way ANOVA followed by Bonferroni post-test (*p ≤ 0.05, **p ≤ 0.01, ***p ≤ 0.0001). (**D–F**) Capacity of recombinant *E. coli* cells expressing MBP alone (Ctrl) or an individual MBP-peptide fusion (peptides P1 to P7) to resist copper as measured by growth in LB broth containing copper sulphate at a final concentration of 4 mM (**D**), 6 mM (**E**), and 8 mM (**F**). Optical density (OD_600_) of each culture grown at 37 °C was recorded every hour, up to 6 h, and with a final reading at 22 h. Data is presented as the mean ± SEM for three independent colonies.

By expressing the *malE*-tagged synthetic degenerate DNA duplex proposed to encode for a bank of metal binding peptides in a harmless non-pathogenic *E. coli*, we aimed to select for the amino acid combinations that possess more significant metal (Cu^2+^) binding coordination site(s). We argued that the ability of bacteria to thrive in the presence of higher copper concentrations could be an indirect measurement of their ability to produce peptides that bind Cu^2+^ ions. Hence, to assess qualitatively the inhibitory copper concentrations resisted by *E. coli* expressing the peptide fusions, we performed initial growth analysis using a copper sulphate gradient agar plate (Fig. 2A). In comparison to non-pathogenic *E. coli* expressing only MBP, bacteria expressing chimeric MBP (cMBP) with peptide, P1 to P7, conferred significantly more protection to toxic levels of copper sulphate (Fig. 2B). Semi-quantification of the bacterial growth appearing on the agar plates revealed that *E. coli* expressing cMBP2 and cMBP3 resisted up to ~ 4 mM copper sulphate (Fig. 2C), whereas cells expressing cMBP4 to cMBP7 resisted up to ~ 2 mM copper sulphate (Fig. 2C). These growth profiles were significantly different from *E. coli* expressing only MBP (Fig. 2B,C). Additionally, cells expressing cMBP1 showed quite low protection with the extent of growth being only marginally better than the MBP control (Fig. 2B,C). Consistent with growth on a copper sulphate gradient plate, cells expressing cMBP1-7 also maintained growth in liquid broth culture in the presence of 4 mM of copper sulphate compared with control bacteria (Fig. 2D). Moreover, growth of cells expressing cMBP3 was noticeably better at this concentration (4 mM). This suggested better counteraction of the toxic effect of copper by these recombinant cells. Further, exposure to higher concentrations of copper sulphate, i.e.: 6 and 8 mM, resulted in cessation of growth for all recombinant bacteria (Fig. 2E,F). Taken altogether, cells expressing cMBP2 to cMBP7 were more resistant to higher concentration of copper sulphate in comparison to the control cells and cells expressing cMBP1. Hence, the peptides P2 to P7 may incorporate copper with appreciable amount, with the most effective being the peptides P2 and P3. Protection of some recombinant bacteria from normally inhibitory copper concentrations could indicate that individual peptides confer to host bacteria the ability to assimilate copper.

### In vitro characterisation of Cu^2+^ binding by cMBP fusions

**Figure 3:**
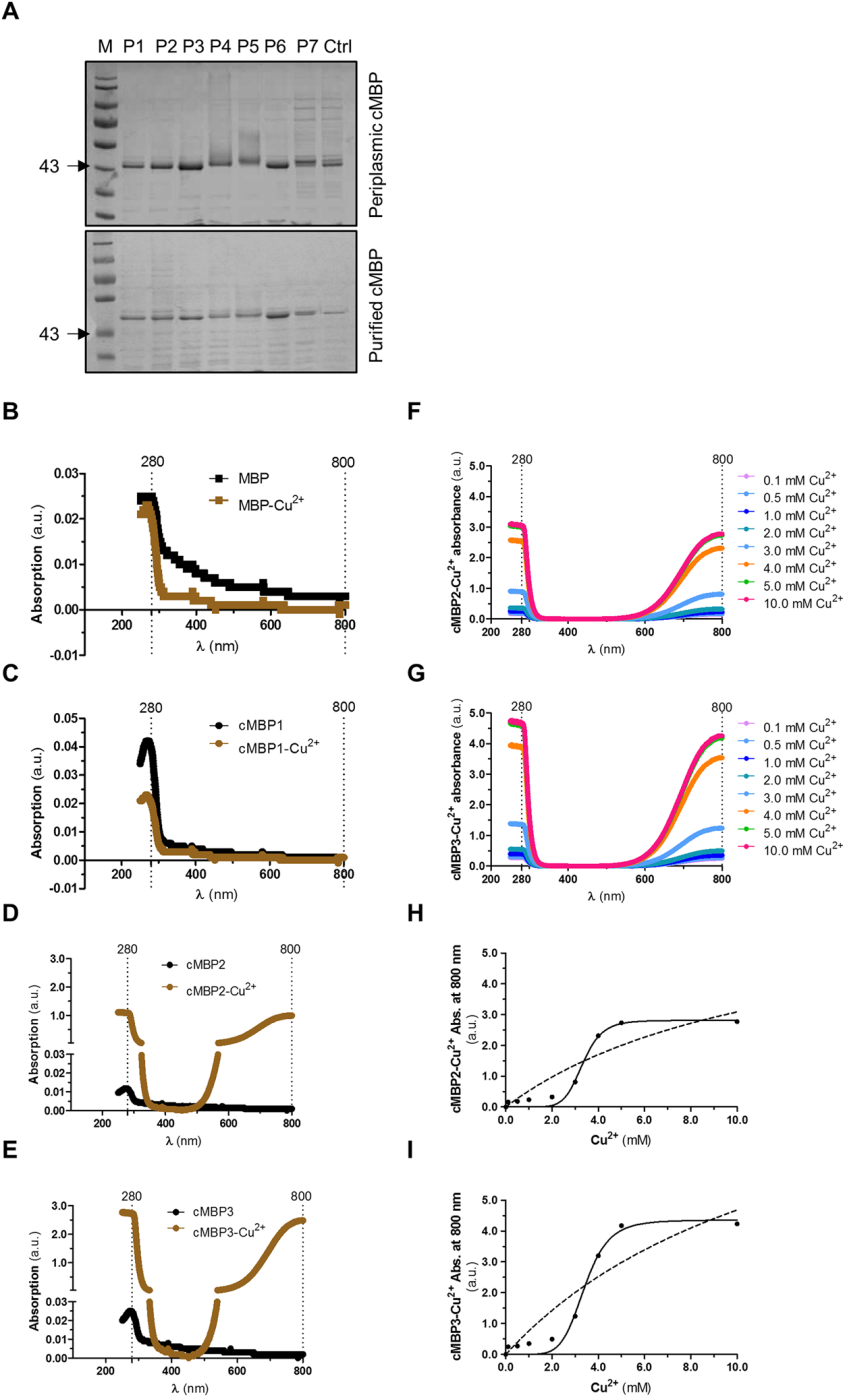
In vitro incorporation of Cu^2+^ by purified cMBP fusions. (**A**) Analysis of crude periplasmic fractions (upper panel) and column-purified periplasmic cMBPs (lower panel). Equal volumes of all samples were fractionated on 10% acrylamide SDS-PAGE followed by coomassie blue staining. Lane 1 indicates the molecular masses of the protein standard (M) in kDa. Lanes 2–8 indicate proteins-expression profiles of recombinant *E. coli* expressing cMBP1 through to cMBP7 (MBP attached with the seven different putative metal binding peptides, P1 to P7). Lane 9 shows recombinant *E. coli* expressing only MBP alone and used as a negative control (Ctrl). Arrows indicated the location of expressed MBP or cMBP1 to cMBP7 (~ 40.8 kDa). A representative gel from three independent biological replicates is shown. Uncropped full length representative gels of SDS-PAGE are added in the Supplementary data as Fig. S2. (**B-E**) UV–VIS spectroscopy of purified recombinant MBP derivatives. Spectra was obtained based upon 152.5 µg of protein in a assay volume of 1 ml of MBP (**B**), cMBP1 (**C**), cMBP2 (**D**), and cMBP3 (**E**) in the absence (black line) or presence of a final concentration of 4 mM CuSO4.5H_2_O (brown line). Absorption peak at or around 280 nm represent level and quality of MBP and cMBP1 to cMBP3 fusions from its aromatic amino acids and peak at or around 800 nm indicate Cu^2+^ incorporation within MBP or cMBP fusions (**F–I**) In-vitro measurement of Cu^2+^ incorporation by cMBP2 and cMBP3. Titration of cMBP2 (F and H) and cMBP3 (**G**,**I**) with 0.1–10.0 mM Cu^2+^ along with associated Michaelis–Menten (dotted line curve) and Allosteric Sigmoid kinetics (solid line curve).

The N-terminal signal peptide of MBP can successfully export fusion chimeras into the periplasm^28^. Localisation into the periplasm allows purification of fusion chimeras with relative ease. Hence, we isolated periplasmic fractions derived from bacterial strains expressing MBP alone or cMBP fusions. These fractions contain the respective protein of interest, confirming their export to the *E. coli* periplasm (Fig. 3A). One-step maltose-affinity chromatography was used to purify MBP with a yield of 107.51 μg/ml and the cMBP fusions to yields of 489.57 μg/ml (for P1), 645.72 μg/ml (P2), 703.45 μg/ml (P3), 478.27 μg/ml (P4), 467.18 µg/ml (P5), 685.36 µg/ml (P6), and 110.12 µg/ml (P7) (Fig. 3A). For in vitro analysis, we used three cMBP fusions – cMBP1, the fusion with the least effective peptide (P1), and cMBP2 and cMBP3, the fusions with the most effective peptides, P2 and P3, respectively.

UV–Vis spectroscopy analysis in the presence of Cu^2+^ ions identified peptide- Cu^2+^complexes only for cMBP2 and cMBP3 fusions, as indicated by absorption peaks at around 800 nm (Fig. 3B–E). Both cMBP2 and cMBP3 in the presence of Cu^2+^ showed a shift in the absorption peak at 280 nm (Fig. 3D,E). This is reflective of efficient copper incorporation by these two peptides. In contrast, cMBP1 (least active peptide) generated UV–Vis spectra similar to the MBP control, regardless of the presence or absence of Cu^2+^ ions (Fig. 3B,C). This indicates negligible incorporation of Cu^2+^ by the cMBP1 fusion. Overall, purified cMBP2 and cMBP3 showed significant copper binding in vitro, and this corroborates protection to toxic Cu^2+^ levels by our recombinant non-pathogenic *E. coli* expressing either cMBP2 or cMBP3.

Employing UV–Vis spectroscopy, we further examined relative copper incorporation by cMBP2 and cMBP3 fusions via a titration experiment (Fig. 3F–I). Successive increments in Cu^2+^ concentration enhanced the intensity of absorption peak at both 280 nm and 800 nm, indicating absorption maxima at our highest tested copper concentration (10 mM) for both cMBP. We used 10 mM copper sulphate as the highest concentration of Cu^2+^ simply because this value is considerably more than the recognised EC_50_ toxic levels of copper in soils. Michaelis–Menten and Allosteric Sigmoid kinetics calculations indicated the cooperativity of cMBP2 with Cu^2+^ is slightly lower than that of cMBP3, in the order of a 1:3 cMBP: Cu^2+^ molar ratio compared to 1:2, respectively (Fig. 3G,H). Altogether, these in vitro assays suggest that cMBP2 and cMBP3 can bind substantial amounts of copper (i.e.: > 4.0 mM).

### Structure modelling and molecular dynamic simulation of the cMBP- Cu^2+^ interaction

**Figure 4:**
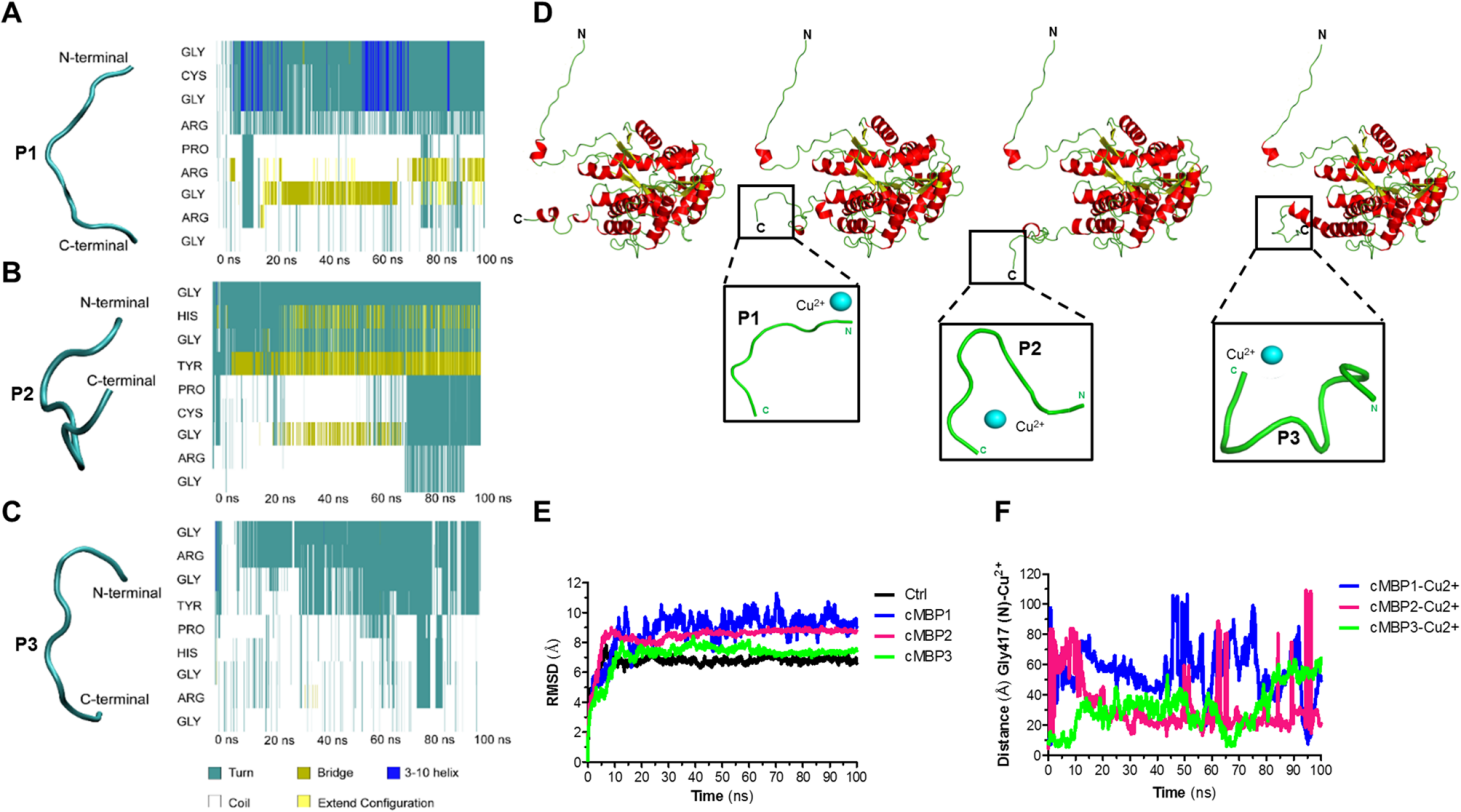
Structure modelling and MD simulation of recombinant fusions. (**A–C**) Secondary structures of standalone peptides P1 (**A**), P2 (**B**) and P3 (**C**) derived after cleavage from the IntFold-modelled full-length structures of cMBP1, cMBP2 and cMBP3, respectively, followed by a 100 ns MD simulation run in the absence of Cu^2+^ ion. Shown is the propensity of the P1 to P3 peptides during 100 ns MD simulation run to form Turn, Bridge, 3_10_-Helix, Coil and extended configuration elements (represented as different colours). Protein snapshot is from the last frame of the simulation. (**D**) PyMOL^56^ alignment of the IntFold-modelled full-length structures show overall conservation of conformation, but with anticipated variation at the respective C-terminus, where the peptide of interest is fused (boxed). A zoomed out version of each peptide with docked Cu^2+^ ion is shown as an inset. (**E**) RMSD values (in absence of Cu^2+^) for MBP alone and the three chimeric fusions, cMBP1 to cMBP3. The X-axis represents the time scale (100 ns) of simulation while the Y-axis is the RMSD values in Angstrom (Α°). (**F**) MD simulations (in presence of Cu^2+^) for MBP alone and the three chimeric fusions, cMBP1 to cMBP3. Interatomic distances between the Nitrogen atom of first Gly residue (for all three peptides) and the Cu^2+^ ion docked to the respective cMBP. The X-axis values represent the time scale, while the Y-axis is the distance in Angstrom (Α°).

We observed that MBP-fused P2 and P3 possess an appreciable amount of copper binding propensity. With the idea to explain favorable Cu^2+^ binding, and whether this binding correlated to peptides possessing a proposed U-shape metal-binding pocket, we used in silico analysis to compare and contrast the potential molecular structure of peptides P2 and P3. We hypothesized a U-shape binding pocket from several known illustrations of active Cu^2+^ binding pockets encased in various different protein families^29^. For comparison, we included the poorest copper binder, MBP-fused P1 peptide. Possible structural features of these peptides were suggested using the structure-modelling predictions and Molecular dynamics (MD) simulation after removing from the respective full-length cMBP model in the absence of Cu^2+^ ion. Peptide P1 appears to lose the helical structure propensity during the simulation, probably because of the inherently unstable 3_10_-helical feature (Fig. 4A). Only the first 4 residues, Gly1 to Arg4, seem to create a Turn-like structure, and the peptide possesses an overall linear conformation (Fig. 4A). In contrast, peptide P2 modelling indicated a possible Turn-like structure from the coil feature through to 70 ns simulation (Fig. 4B). Interestingly, peptide P3 modelling predicted two turns and the absence of any extended configuration that might be a basis for better accommodation of the Cu^2+^ ion (Fig. 4C). Moreover, only in P2 and P3 models was the conserved Pro residue at position 5 contributing to the putative Turn-like structure. Collectively, only in models of P2 and P3 was there suggestion of a stable loop conformation. These findings are consistent with our initial hypothesis that a U-shaped structure would favour better Cu^2+^ ion coordination, as inspired from known active-site Cu^2+^ binding configurations^29^. To our knowledge, this is the first report of a synthetic ‘degenerate’ DNA motif of 30 bp used to liberate a bank of semi-random peptides with potential to generate U-shaped conformations that coordinate copper binding. Further, the predicted full-length modelled structures upon interaction with Cu^2+^ ion were similar at the N-terminal end reflecting the MBP molecule, and with some differences towards the C-terminal end reflecting contributions from the fused peptide (Fig. 4D). Critically, the peptides of cMBP2 and cMBP3 fold with a hairpin secondary structure that could help in accommodating the metal ions^30^ In contrast, the peptide of cMBP1 forms a non-hairpin like secondary structure (Fig. 4D)*.*

Subsequently, simulated full length MBP and cMBP structures in the absence of Cu^2+^ ions were analysed under physiological conditions. Simulation of these modelled structures indicate that in the absence of Cu^2+^ ion, the cMBP3 structure is dynamically more stable given that the root mean square deviation (RMSD), a dynamic stability index, is lower for the structures of cMBP1 and cMBP2 when simulated with identical parameters (Fig. 4E). The control MBP structure shows lowest RMSD value, indicating it to be dynamically very stable (Fig. 4E). However, we caution that this low RMSD value can be partly attributed to MBP alone having a shorter sequence. On the other hand, the greater dynamic instability of cMBP1 corroborates the lower Cu^2+^ binding potency measured both in vitro and in vivo (see Figs. 2 and 3). Thus, we surmise from these MD simulations in the absence of Cu^2+^ ion that cMBP2 and cMBP3 structures are dynamically more stable than cMBP1, and this may partly explain their enhanced ability to bind Cu^2+^ both in vitro as well as in vivo.

Intriguingly in the presence of Cu^2+^, throughout the entire simulation run the molecular interaction distance of Cu^2+^ ion with the N-atom of the first Gly1 residue in all three chimeric peptides routinely indicated a lower Å distance between the ion and cMBP2 or cMBP3 compared to the ion and cMBP1 (Fig. 4F). The cMBP2- Cu^2+^ interaction, and to a lesser extent the cMBP3- Cu^2+^ interaction, appears to be more stable at least until 50 ns. On the other hand, the cMBP1- Cu^2+^ interaction appears very unstable considering the observed higher distance of cMBP- Cu^2+^ interaction and many fluctations in simulation (Fig. 4F). Remarkably, the cMBP3- Cu^2+^ interaction generated very low distance until 10 ns, implying that the docked Cu^2+^ ion could remain in the binding pocket. In contrast, the docked Cu^2+^ ion appeared to leave the binding pocket of cMBP1 and cMBP2 within 3 ns of stimulation. Hence, MD simulations predict that a Cu^2+^ ion binds preferentially to cMBP2 and cMBP3, and not to cMBP1.

### In silico prediction of metal ion interaction sites

**Figure 5:**
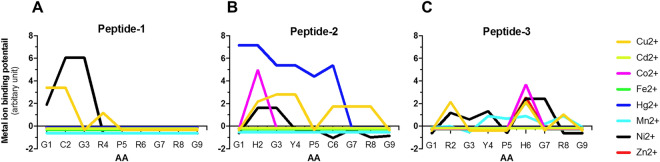
In silico prediction of metal ion binding potential by the recombinant fusions. Shown is the metal ions binding potential score per amino acid (AA) residue of the C-terminally fused respective peptide (**A–C**) of chimeric cMBP1 to cMBP3 for eight different divalent transition metals as predicted by the MIB server. Metal ion binding potential score for respective entire chimeric protein is shown in Fig. S3.

To have any relevance to environmental clean-up applications, it is important to appreciate the metal binding affinity and specificity of our engineered MBP-peptide chimeras. Experimental identification of metal binding sites within poly(peptides) can be challenging as it requires expensive and specialized techniques, such as NMR spectroscopy^31^, absorption spectroscopy^32^, metal-affinity column chromatography and electrophoretic mobility shift assay^33^. In contrast, bioinformatic analysis of metal ion binding sites capatilises on the vast information accumulated in public database to generate rapid, accurate and reliable predictions. We used the MIB server^34^ to assign predictions for metal binding affinity because it has proven reliability for predicting binding sites for up to 12 metal ions. The prediction of metal ion binding is based upon a fragment transformation method^35^, where each amino acid within a given peptide was considered as an individual structural unit in order to search for potential metal ions against a reference list of ~ 40,000 metal binding poly(peptides) in the Protein Data bank^36^. Of eight tested divalent metal ions, only Cu^2+^ and Ni^2+^ ions were predicted to engage with peptide P1 (Fig. 5A). Residue Cys2 displayed predicated affinity for both ions, while residue Arg4 only for Cu^2+^ ion. The modest metal binding prediction mirrors the in vitro results of negligible binding of cMBP1 with Cu^2+^ ion (see Figs. 2 and 3). The peptide P2 displayed a predicted metal ion binding affinity toward the ions Cu^2+^, Ni^2+^, Co^2^ and Hg^2+^ (Fig. 5B). Affinity for Cu^2+^ ion was found at residues His2, Tyr4, Cys6 and Arg8, indicating a potent Cu^2+^ coordination within this peptide. The peptide P3 displayed a predicted broad metal ion binding affinity towards the ions Cu^2+^, Co^2+^, Mn^2+^, Ni^2+^and Zn^2+^ (Fig. 5C). Within peptide P3, the residues Arg2, His6 and Arg8 appear to have a marked metal ion binding potential for Cu^2+^ ion. The His6 residue also displayed predicted affinity for Mn^2+^, Ni^2+^ and Zn^2+^ ions. Collectively, the fused peptides appear to possess a broad metal ion binding affinity in the order cMBP3 > cMBP2 > cMBP1, corroborating the degree of copper binding achieved by the three peptides (see Figs. 2 and 3).

## Discussion

Copper is an essential trace element for living organisms and their ecosystems, but at higher concentrations it is toxic. Anthropogenic activities have elevated copper concentrations well beyond the current environmental quality standard^1,6–13,37^. This is concerning given that copper pollution of the environment is commonplace. Hence, there is an urgent need to develop systems for the efficient, cost-effective and environment friendly remediation of copper.

With a view to develop a technology to overcome copper pollution, we constructed a biological system intended for the eventual bioaccumulation of Cu^2+^ ions. A harmless non-pathogenic *E. coli* strain DH5ᾳ was engineered to express a small bank of semi-random peptides fused at their C-termini to MBP harboured within the IPTG inducible plasmid, pMAL-p2x. Under the laboratory conditions tested, recombinant *E. coli* expressing six of the seven MBP-peptide chimeras acquired tolerance to high concentrations of copper. Among these, bacteria expressing either cMBP2 or cMBP3 were particularly tolerant to higher Cu^2+^ concentrations. In this study, we did not perform Cu^2+^ uptake experiments to demonstrate Cu^2+^ accumulation by peptide-expressing bacteria. However, we showed clearly that the extent of copper tolerance by cultured non-pathogenic *E. coli* bacteria expressing either cMBP2 or cMBP3 was consistent with in vitro incorporation of copper by the purified cMBP2 and cMBP3 variants.

This study was not intended to be an exhaustive screen of Cu^2+^-ion binding peptides. Of the many recovered white colony transformants from the original transformation plated on selective agar plates supplemented with X-gal and IPTG, sequencing of a selection of these revealed seven unique DNA sequences, each encoding for a subtly different peptide. Had we continued to screen, we may have recovered other unique sequences. However, after examining the positive effect of expressing this random selection of seven peptides – P1 to P7 – in *E. coli* grown in the presence of a toxic Cu^2+^ ion gradient, we considered that these were a sufficient representation to establish this proof-of-concept study.

Noted published studies have shown enhancement of Cu^2+^ absorption up to 32-fold using engineered bacteria expressing metal binding peptides. For example, Pazirandeh and colleagues engineered *E*. *coli* cells expressing a triplet of small peptides, ^N^CGCCG^C^ in fusion with periplasmic MBP, but reported removal of less than 40% of a modest 5 µM copper ions from a liquid medium after 1 h incubation^24^. Moreover, Ueki and co-authors reported that *E*. *coli* BL21 cells expressing two vanabins, which are small cysteine-rich proteins distantly related to metallothioneins, in fusion with periplasmic MBP, absorbed about 70% of a modest 10 µM copper (II) ions from an aqueous medium^27^. Although we did not perform Cu^2+^ adsorption experiments in this study, bacteria expressing some of our peptides conferred much higher resistance to Cu^2+^ ions. These observations correlated to in vitro and in silico Cu^2+^ incorporation studies. In fact, measurements produced herein suggest that our biological system has the potential to assimilate copper at levels almost 160-fold higher than the recognised EC_50_ toxic levels of copper in soils^11^.

A natural progression of this work is therefore to express our copper-binding peptides in *E. coli* strains with improved copper sensing capacity. Hence, the recent report by Ravikumar and colleagues is significant. These authors describe an *E. coli* system based upon a small copper binding peptide, ^N^SPHHGGW^C^ fused to OmpC, and with its expression controlled by the extracellular copper sensing two-component system, CusSR^38,39^. Assembly of this genetic circuit improved copper adsorption affinity up to 92.2 μM^38^. Given this, it is possible that incorporation of our hyper copper-sequestering peptides (P2 and P3) with OmpC and expressed under the control of an intact CusSR two-component system might generate a strain with improved environmental copper sensing ability in association with hyper copper bioaccumulation capacity. It is also relevant that Wang and co-authors recently engineered a *copA* and *cueO* mutant of *E. coli* that was capable of detecting 0.01–25 µM copper and efficiently adsorbing 125 µM copper ions from aqueous solutions^40^. Our hyper copper-sequestering peptides could easily be incorporated into the genetic system of Wang and co-authors^40^ to generate an *E. coli* strain with capacity for simultaneous detection and removal of copper in a temperature dependent manner. Thus, options exist to refine our system to enhance copper removal capacity and avoid reliance on artificial induction by expensive chemical synthetics.

Interestingly, structural modelling by IntFOLD coupled to protein dynamics assessment by RMSD and MD simulation indicated that the peptide sequence when appended to MBP negatively influenced the protein stability in the order of cMBP3 < cMBP2 < cMBP1. Although this would need to be confirmed by biophysical experiments, such as by performing circular dichroism studies, it is a possibility that enhanced peptide P3 and P2 stability maximises their apparent Cu^2+^ binding affinity as measured experimentally in both in vitro and in vivo contexts. As protein structures are unrestrained and free to move in all directions during the period of simulation, bound ion releases from the binding site. The rate of ion release is based on the flexibility of the binding site, which in turn infers information about the strength of the interaction^41^. MD simulation studies are therefore commonly employed to access this information^30^. A well-established example of this is with the assessment of Na^+^ and Cl^-^ ion shuttling on the surface of the S6 ribosomal protein^42^. When applied to our peptides, 100 ns MD simulations predicted release rates of Cu^2+^ ions that in turn indicated superior ion binding in the order of cMBP3 > cMBP2 > cMBP1. This makes sense considering the prediction of a distinct U-shaped turn for P2 and P3, compared to an overall linear conformation for P1. We believe that the U-shape could help P2 and P3 peptides to accommodate the Cu^2+^ ion. Interestingly, the guanidine group of arginine residues within both P2 (^N^GHGYPCGRG^C^) and P3 (^N^GRGYPHGRG^C^) appeared to anchor Cu^2+^ ions in a stable metal-peptide complex. Such a role for arginine has been suggested previously^43^. Finally, proline residues are favourable for the turn formation^44^. Proline was engineered at position 5 of each peptide. However, its presence in P1 was unable to promote loop formation. Perhaps arginine residues on either side of this proline might be the reason that stable loop conformation could not be formed as previously shown by the role of the arginine in increasing the unfolding rate^45^.

Computational predication of metal ion binding affinity is a powerful recent development. Compared to traditional specialised methods that are often expensive and tedious, such as NMR, chromatography, gel electrophoresis and spectroscopy^31,32,46,47^, available in silico prediction tools now enable an accurate, fast and efficient approach to identify metal ion binding affinity and specificity^48^. Corroborrating data obtained with Cu^2+^ in both in vitro and in vivo experimental contexts, the bioinformatic MIB server^34^ predicted broad range metal ion affinity in the order of cMBP3 > cMBP2 > cMBP1. Moreover, the MIB server indicated affinity for the Cu^2+^ ion was coordinated through histidine, tyrosine, cysteine and arginine residues, consistent with information inferred from our structural modelling and protein dynamic simulations. The histidine residue is of particular importance, not only for enabling broad binding specificity to multiple metal ions, but also for establishing the extent of the binding affinity. In this regard, the positioning of histidine within the peptide sequence seems to have consequence for both specificity and affinity given that it is positioned at residue 6 in the most potent peptide, P3, while positioned at residue 2 in the somewhat inferior performed peptide, P2. Significantly, there is precedent for a role of histdine residues in influencing copper-binding site behavior as implied by mutagenesis and catalytic activity studies of human tyrosinase^49^.

In summary, a combinatory approach of synthetic biology with bacterial physiology enabled the generation of a small bank of Cu^2+^ assimilating peptides. When expressed by *E. coli* DH5α, the peptide fusions – cMBP2 and cMBP3 – could bind Cu^2+^ ions with higher capacity than previously reported. This includes being almost 32-fold higher than an *E. coli* strain engineered to sequester Cu^2+^ from Chinese wastewater^40^, and about 160-fold higher than the recognised EC_50_ toxic levels of copper in soils^11^. Thus, we demonstrate a concept for potential development of a cost-effective and environment friendly biological system to counteract copper pollution. This system is also open for possible broad-spectrum use to sequester other hazardous heavy metal contaminants or might even be applied to the bio-mining of rare metals.

## Materials and methods

### Designing of synthetic degenerate DNA oligonucleotides

In order to craft a bank of metal binding peptides, a previously described approach^26^ was employed with modifications. Briefly, two synthetic degenerate DNA oligonucleotides (30 nt long), 5′-AATTCGGT**YR**TGGC**YR**CCCG**YR**TGGC**YR**CGGTTGAG-3′ (oligo 1) and 5′-GCCA**RY**ACCG**RY**GGGC**RY**ACCG**RY**GCCAACTCCTAG-3′ (oligo 2) were designed and purchased from Eurofins MWG Operon, Ebersberg, Germany. Both oligos were complementary to each other and annealed (in-house) together to form a duplex ‘degenerate’ metal binding DNA motif that would have potential to encode for small ‘degenerate’ peptides of 9 amino acids in length. All peptides were to initiate with Gly and terminate with a stop codon. These peptides would have random distribution of potent metal binding amino acids Cys, His, Arg, and Tyr at position 2^nd^, 4^th^, 6^th^ and 8^th^ with an invariant Gly spacers and Pro hinge at the centre (5^th^ position) to form a hypothesised U-shaped pocket to occupy metal ion(s).

### Cloning of synthetic degenerate dsDNA motif

Cloning of metal binding synthetic degenerate dsDNA motif in-frame at the 3′-end of *malE* (encoding MBP) within a commercially available pMAL-p2x plasmid (New England Biolabs, Ipswich, MA, USA) was carried out according to the protocol provided by the manufacturer (New England Biolabs). Briefly, the pMAL-p2x plasmid DNA was digested with EcoRI and BamHI and purified from an agarose gel matrix using the GeneJET Gel Extraction Kit (Thermo Fisher Scientific, Waltham, MA, USA). Equal volumes of the two synthetic degenerate ssDNA motifs were annealed, and an aliquot ligated overnight into the purified linearised pMAL-p2x plasmid using T4 DNA ligase. The ligation mixture was transformed into the in-house prepared chemically competent *E. coli* DH5α bacterial cells [Genotype: F^–^ φ80*lac*ZΔM15 Δ (*lac*ZYA-*arg*F) U169 *rec*A1 *end*A1 *hsd*R17 (r_K_^–^, m_K_^+^) *pho*A *sup*E44 λ^–^ *thi*-1 *gyr*A96 *rel*A1] and plated onto selective Luria Bertani (LB) agar containing ampicillin (50 μg/ml), 5-bromo-4-chloro-3-indolyl-β-D-galactopyranoside (X-gal) (40 μg/ml), and Isopropyl β-D-1-thiogalactopyranoside (IPTG) (100 μM). After overnight growth, seven white colony transformants that contained clones likely expressing metal binding degenerate peptides at the C-terminus of MBP, assigned as Chimeric MBP (cMBP) were randomly picked for plasmid DNA isolation and subsequent sequence analysis.

### Deciphering recombinant plasmid DNA sequences

Using the Qiagen miniprep kit (Qiagen, Hilden, Germany), plasmid DNA was purified from seven selected individual colonies of *E. coli* strains that express cMBP under tight control of IPTG-inducible P_tac_ promoter^50,51^. Purified plasmids DNA were sequenced using M13-47 sequencing primer (NEB # S1224S: 5′-CGCCAGGGTTTTCCCAGTCACGAC-3′). All sequence variants encompassing *malE*-fused metal binding degenerate DNA motif that were translated by the ExPASy translation tool (https://www.expasy.org/) and a consensus signature of degenerate peptides predicated using the WEBLOGO server, https://weblogo.berkeley.edu/logo.cgi.

### Growth of recombinant non-pathogenic *E. coli* on LA agar supplemented with Cu^2+^

Resistance to copper of recombinant *E. coli* strains expressing sequence confirmed cMBP (in the periplasm) was initially assessed in the presence of copper sulphate (CuSO_4_.5H_2_O). A gradient (0 to 10 mM) of copper sulphate was established in sterile square petri plates. Molten LB agar (25 ml), supplemented with ampicillin (50 μg/ml), IPTG (0.1 mM) and CuSO_4_.5H_2_O (10 mM) was poured diagonally into the plates under sterile conditions. Following solidification, a further 25 ml molten LB supplemented only with ampicillin (50 μg/ml) and IPTG (0.1 mM) was overlaid onto the gradient of copper sulphate. A standardised inoculum (10 µl of 0.1 OD_600nm_) of overnight grown (in selective LB liquid broth containing ampicillin (50 μg/ml) and IPTG (0.2 mM) at 37 °C with agitation) bacterial culture was aseptically streaked directionally from predictable lower to higher concentration of copper sulphate. All streaked plates were incubated at 37 °C overnight. Bacterial growth was analysed toward higher concentrations of copper sulphate and compared with the growth of *E. coli* expressing MBP alone.

### Growth of recombinant non-pathogenic ***E. coli*** in LB broth supplemented with Cu^2+^

A pre-culture inoculum of recombinant *E. coli* strains expressing either MBP alone or sequence confirmed cMBP was prepared by growing (at 37 °C with agitation overnight) into selective LB liquid broth (5 ml), containing ampicillin (50 μg/ml) and IPTG (0.2 mM). Next day, 15 ml selective LB broth was inoculated with a standardised number of cells (OD_600_ ~ 0.02) from overnight cultures and in the presence of 4, 6, and 8 mM copper sulphate and incubated at 37 °C with agitation. Optical density (OD_600_) was measured in hourly intervals up until 6 h and with a final reading at 22 h.

### Periplasmic fractions of recombinant non-pathogenic *E. coli*

Periplasmic fraction from the genetically engineered *E. coli* strains was prepared according to the protocol provided by the manufacturer (New England Biolabs protocol) with some modifications. Briefly, single fresh colonies of *E. coli* expressing either MBP alone or cMBP fusions were inoculated into 5 ml selective LB broth (supplemented with 2.0 mg/ml glucose and 50 µg/ml ampicillin) and grown overnight at 37 °C with agitation. Next day, 200 ml selective LB broth was seeded with a standardised number of cells (OD_600_ ~ 0.02) and grown until OD_600_ ~ 0.5 and then induced with IPTG (0.3 mM final concentration) and grown for a further 2 h at 37 °C with agitation. Cells were harvested by centrifugation at 4,000 × *g* for 20 min at 4 °C. Pelleted cells were mixed with 25 ml Osmotic shock buffer (30 mM Tris–HCl; pH 8.0, 20% sucrose, 1 mM EDTA). Resulting cell-suspensions were incubated at 37 °C for 7 min with shaking. Following centrifugation at 8,000 × *g* for 10 min at 4 °C, the supernatants were discarded, pelleted cells were mixed with 25 ml ice-cold 5 mM MgSO_4_ and incubated at 4 °C for 10 min with gentle agitation. Ice-cold suspensions were centrifuged, and the supernatants were recovered as periplasmic fractions. Fifty µl from each fraction was mixed with sample buffer (100 mM Tris–HCl; pH 6.8, 4% SDS, 20% glycerol, 294 mM β-mercaptoethanol, 25 mM EDTA, 0.04% Bromophenol blue), boiled at 95 °C for 5 min, and 10 µl of each sample run on 10% acrylamide SDS-PAGE followed by coomassie staining. The remaining periplasmic fractions were used for purification.

### Purification of MBP and cMBP fusions from the periplasmic fractions

The MBP alone and the cMBP fusions were purified by one-step Maltose affinity chromatography according to the protocol provided by the manufacturer (New England Biolabs protocol) with some modifications. Briefly, recovered periplasmic fractions were mixed in 20 ml of amylose-column buffer (20 mM Tris–HCl, 200 mM NaCl, 1 mM EDTA and 10 mM β-Mercaptoethanol; pH 7.45), supplemented with 1 × cOmplete protease inhibitor cocktail (Sigma-Aldrich, Gillingham, Dorset, UK). Columns (2.5 × 10 cm) were packed with amylose resin, equilibrated with 100 ml of column binding buffer, and then loaded with periplasmic fraction mixtures at a rate of 0.5 ml/min. Flow-through was collected and the columns washed with 100 ml of column buffer at 1 ml/min. Unbound and washed samples were collected. Amylose-resin bound with MBP or the cMBP fusions were eluted at a rate of 0.5 ml/min with 10 mM maltose containing column buffer. Ten fractions (1 ml each) were collected and analysed by SDS-PAGE. Fractions containing most of the MBP or cMBP protein were pooled and concentrations of each was measured using Protein Kit (Bradford Method) reagent solution. An equal volume (25 µl) of the purified-pooled fraction containing either MBP or a cMBP fusion was mixed with sample buffer and analysed by SDS-PAGE.

To de-salt and exchange the buffer of the eluted pooled fractions of MBP and cMBP, PD-10 desalting columns prepacked with Sephadex G-25 medium were used. Briefly, columns pre-equilibrated with 25 ml of elution buffer (20 mM Tris–HCl, pH 7.45), were loaded with 2.5 ml of the respective protein fraction. Bound protein was eluted in 1 ml fractions with 4 ml of elution buffer. An equal volume from each fraction was analysed by SDS-PAGE. Those fractions containing most of the eluted MBP or cMBP fusion protein was pooled together and stored at 4 °C until further biochemical analysis.

### In vitro quantification of Cu^2+^ incorporation by cMBP fusions

A JASCO-V560 UV–Vis Spectrophotometer was used to quantitate Cu^2+^ ions incorporation into the MBP and cMBP1 to cMBP3 fusions. Briefly, an equal volume of purified-desalted periplasmic samples containing 1 × cOmplete protease inhibitor cocktail (Sigma-Aldrich, Gillingham, Dorset, UK) was mixed with a final concentration of 4 mM CuSO_4_.5H_2_O in a total sample volume of 1 ml. Samples were incubated overnight at room temperature and UV–Vis spectra of each was recorded at 190 to 900 nm wavelength. Additionally, cMBP2 and cMBP3 samples (also contain 1 × cOmplete protease inhibitor cocktail) were incubated overnight at room temperature with 0.1 to 10 mM CuSO_4_.5H_2_O and on the following day their UV–Vis spectra recorded. We used 10 mM copper sulphate as the highest concentration of Cu^2+^ because this is considerably more than the recognised EC_50_ toxic levels of copper in soils. Peak absorption values at 800 nm peaks (representing Cu^2+^ bound cMBP) were further analysed by well-known Michaelis–Menten and Allosteric Sigmoid kinetics in order to demonstrate Cu^2+^ ions cooperativity to cMBP2 and cMBP3 fusions.

### Structure modelling and MD simulation

A homology structure-modelling server, IntFOLD^52^ was used to generate the model structures of MBP alone, cMBP1, cMBP2 and cMBP3. Best quality modelled structure of each was selected and were subjected to MD simulation without bound ions. The modelled proteins were solvated with a water cubic box of appropriate dimensions as per the size of protein. Counter ions were added to compensate the charges in the system. Solvated protein structurers were minimised and equilibrated for 500 picoseconds^52^ each. Further, these structures were subjected to production run of 100 nano seconds (ns) of molecular dynamics (MD) simulations using GROMACS-2019 program by utilising GROMOS96 54a7 force field^53^ for protein, water and ions. The protein dynamic was evaluated by root mean square deviation (RMSD) method.

Furthermore, each modelled structures was brought into the AutoDock^54^ environment and one Cu^2+^ ion was docked into the fused peptide portion of each cMBP. The docked poses were subjected to run for 100 ns MD simulation by applying similar parameters as described above. Interatomic distances were calculated for the N-atom of Gly417 (G1 in the peptide). Secondary structures of the fused peptides were calculated (in absence of Cu^2+^) from the reterieved MD simulations, using the Timeline plugin in VMD-1.9.2 software^55^.

### Predictions of metal ions binding affinity

Metal ions binding affinity of each structure was predicted by MIB server^34^. This program uses fragment transformation method^35^ and combines the structural and sequence information to search for local metal interaction sites in the protein. Metal binding affinity for MBP and cMBPs structures was calculated for 8 different types of metal ions with default input parameters.

### Statistical analysis

Statistical analyses were performed using GraphPad Prism statistical software (V8.2.0; San Diego, CA, USA). One-way ANOVA followed by Bonferroni post-test was performed to examine for differences among the various strains and growth conditions. Differences with a value of *p* ≤ 0.05 were considered statistically significant. The densitometric analysis of MBP, MBP-peptide fusion proteins and copper sulphate linear gradient plate was performed using ImageJ 1.48v (NIH).

## Supplementary information

Supplementary Information.

## Data Availability

The datasets analysed during the current study are available from the corresponding author on reasonable request.
