# Supplementary material for: Bioengineering of non-pathogenic Escherichia coli to enrich for accumulation of environmental copper
Source: Sci Rep. 2020 Nov 23;10:20327. doi: 10.1038/s41598-020-76178-z (PMC7683528; doi:10.1038/s41598-020-76178-z)
Supplement: Supplementary file 1 — Supplementary Information. [file 41598_2020_76178_MOESM1_ESM.docx]

**Bioengineering of non-pathogenic *Escherichia coli* to enrich for accumulation of environmental copper**

Dharmender K. Gahlot^1,2,*^, Nayyer Taheri^2^, Dhani Ram Mahato^3^, Matthew S. Francis^2^

^1^Department of Biology, University of York, Wentworth Way, York, YO10 5DD, United Kingdom

^2^Department of Molecular Biology, Umeå University, 90187 Umeå, Sweden

^3^Deartment of Chemistry, Umeå University, 90187 Umeå, Sweden

^*^Address correspondence to Dharmender K. Gahlot, E-mail: [dharmender.kumar@umu.se](mailto:dharmender.kumar@umu.se), Tel: +46-76 751 63 28

Running title: Copper accumulation by bioengineered *E. coli*


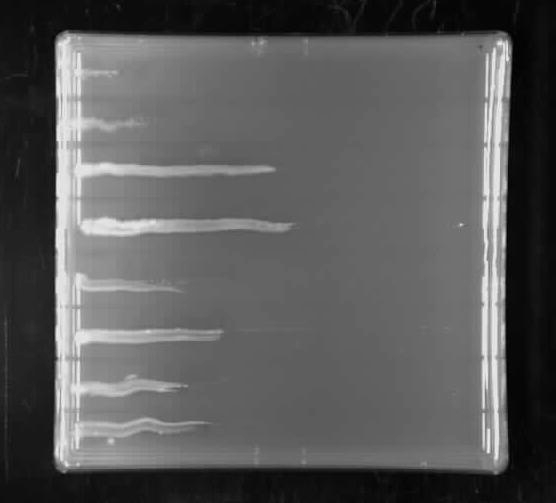


**Ctrl P1 P2**

**P3 P4**

**P5**

**P6**

**P7**


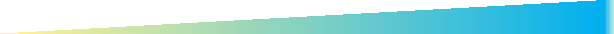


# 0 CuSO4 gradient

**10 mM**

**Figure S1.** Original plate for Figure 2B. Representative gradient plate of three

independent experimental sets. Same plate image is shown in figure 2B.

**kDa M P1 P2 P3 P4 P5 P6 P7 Ctrl -**

**170**


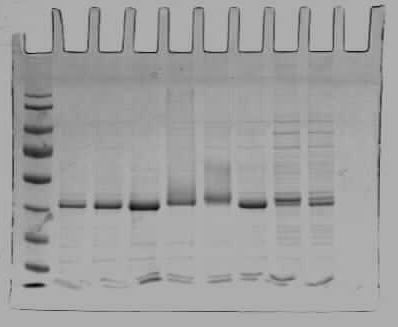


**130**

Periplasmic cMBP

**95**

**72**

**55**

**43**

**34**

**26**

**17**

**170**


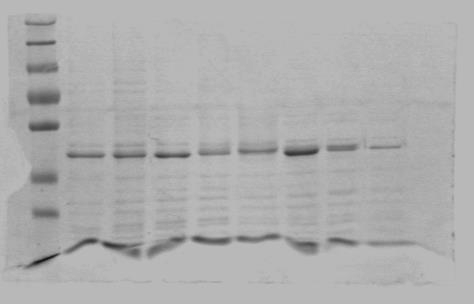


**130**

**95**

**72**

Purified cMBP

**55**

**43**

**34**

**26**

**17**

**Figure S2.** Original gel image for figure 3A. SDS-PAGE of periplasmic fractions and column-purified periplasmic cMBP. Gel inside dotted square is shown in figure 3A.

**A C**

**B D**

**Figure S3.** Metal ions binding potential of Ctrl and cMBP1-3 fusions, predicted by MIB server. Metal ions binding potential score per amino acid (AA) residue of the modelled structure of MBP Ctrl and chimeric MBP1-3 (B-D) is shown with eight different divalent transition metals. A zoomed in version of cMBP1-3 (B-D) C-terminal, encompassing peptide 1-3, is represented in Fig. 5.
